# Interrater and intrarater reliability and minimal detectable change of the Wisconsin Gait Scale when used to examine videotaped gait in individuals post-stroke

**DOI:** 10.1186/s40945-015-0011-z

**Published:** 2015-10-05

**Authors:** Robert Wellmon, Amy Degano, Joseph A. Rubertone, Sandra Campbell, Kelly A. Russo

**Affiliations:** 1 Institute for Physical Therapy Education, Widener University, One University Place, Chester, PA USA; 2 Drexel University, College of Nursing and Health Professions, Philadelphia, PA USA; 3 Inova Mount Vernon Hospital, Alexandria, VA USA

**Keywords:** Gait, Stroke, Outcome assessment, Reliability, Minimal detectable change, Wisconsin gait scale

## Abstract

**Background:**

Often, interventions targeting the kinematic and temporal and spatial changes in gait commonly seen after a stroke are based on observations of walking. Having the capacity to objectively identify such changes and track improvements over time using reliable and valid measures is important. The Wisconsin Gait Scale (WGS), which is comprised of 14 items, was developed specifically to examine and document gait changes occurring after a stroke. The purpose of the study was to explore the interrater and intrarater reliability and minimal detectable change (MDC) of the WGS when used by physical therapists to examine gait in adults post-stroke.

**Methods:**

Fourteen physical therapists from 3 different acute inpatient rehabilitation centers rated videotapes of the gait of 6 adults post-stroke using the WGS. To minimize subject variability from fatigue, videotapes created by using 4 cameras provided right and left lateral, anterior, and posterior views of walking on a level surface. One complete ambulation trial from each subject post-stroke, which included 4 views of the same ambulation trial, was examined by the licensed physical therapists using the WGS. An opportunity was provided to review the tool and a practice trial was performed using an additional videotape not included in the analysis. Gait was examined on 2 different occasions separated by a period of approximately 21 days to minimize the effects of recall bias. Intraclass Correlation Coefficients (ICC) were used to examine the interrater and intrarater reliability of the WGS.

**Results:**

Interrater (ICC = 0.83) and intrarater (ICC = 0.91) reliability were both good. The standard error of the measurement (SEM) was 1.47 and the MDC_95_ was 4.24. There was no statistically significant difference between the scores on the WGS when comparing the 2 different sessions.

**Conclusions:**

The WGS shows promise as an instrument that can make observational gait analysis more reliable. High intrarater reliability and low SEM suggests that the WGS is stable when administered across multiple sessions by the same rater. The ICC for interrater reliability was also good, which suggests that multiple examiners can effectively use the instrument. With minimal training, the physical therapists in the study were able to produce highly reliable results using the WGS to objectively document gait dysfunction.

## Background

Gait disturbances, which are common after a stroke, lead to activity limitations and contribute to participation restrictions [1]. Activity limitations arise due to changes in walking kinematics and kinetics that reflect deficits in motor control associated with altered patterns of muscle recruitment, perceptual motor changes, sensory loss, decreased flexibility and alterations in tone [2–5]. When comparing the hemiparetic side to the unaffected side, temporal and spatial asymmetries in a number of gait measures are frequently observed [6]. Attempts at limiting weight bearing on the paretic limb leads to inequalities in step length [7]. Compared to the unaffected side, the paretic limb single stance time is shorter [7]. The base of support during double stance increases and difficulty clearing the foot on the paretic side during swing can occur [8]. Alterations in trunk alignment and decreased hip and knee flexion and extension are seen [9]. Collectively, these changes limit how fast and far the individual post-stroke can walk and increase both the energy requirements of ambulation and fall risk [6, 10–14].

Appropriately targeting interventions to treat gait dysfunction is an important part of the post-stroke rehabilitation process [15, 16]. Monitoring changes in gait kinematics may help identify the degree of improvement and predict future outcome, assist with treatment planning and allow monitoring the effectiveness of interventions implemented [17–21]. While 3-dimensional kinematic analysis and instrumented approaches provide the best understanding of gait deficits [22], the cost, time requirements and expertise necessary to interpret the findings limit implementation in routine clinical practice. Thus, observational gait analysis (OGA) is commonly employed in daily clinical practice [23]. OGA requires minimal time and expense, and there is no equipment required. However, concerns exist regarding the reliability and validity of OGA. Poor to moderate reliability has been reported, which may be due in part to examiner skill and the lack of a systematic approach to examine gait [24–27]. The data generated can be subjective, depend on examiner skill and lacking in the capacity to detect change over time [24–27].

Standardizing OGA by using forms or standardized instruments that help organized the examination process or observations in a systematic manner can begin to address some of the concerns related to reliability and validity [25, 27, 28]. There are number of instruments available that can help improve the reliability of OGA, such as the Rancho Los Amigos Observational Gait Analysis Scale [29, 30]. While seemingly widely used in practice [30], the Rancho Los Amigos Observational Gait Analysis Scale was not specifically designed to capture the gait kinematic changes seen after a stroke and its use has not been investigated in individuals post-stroke [31]. There are limited number of instruments designed to specifically examine gait in individuals post-stroke [31]. Of those that are available, the Wisconsin Gait Scale (WGS) is a promising stroke-specific activity measure developed to objectively quantify changes in gait kinematics, and spatial and temporal parameters known to adversely affect walking function [32, 33]. The 14-item instrument examines alterations in paretic limb stance time and step length, base of support during double stance, capacity to perform a weight shift and place weight onto the paretic limb during stance, willingness to place weight on the paretic limb at loading response and the capacity to achieve a heel strike, toe clearance and knee flexion during swing, and hip extension at terminal stance. These are common gait parameters that are altered after a stroke [6, 34].

While the WGS has been used in a number of randomized clinical trials demonstrating improvements in patient function for walking [35–37], there is limited research exploring its psychometric properties [31]. In a sample of individuals with chronic stroke, moderate interrater reliability (Kendal Tau-B = 0.44–0.85) was reported for 2 raters by Rodriquez and colleagues [32].

Yaliman and colleagues examined intrarater and interrater reliability in a sample with chronic stroke using a version of the WGS that was translated into Turkish [38]. Chronbach’s alpha across the administration of the instrument on 2 separate occasions, 2 days apart indicated excellent internal consistency for the items included in the instrument. Across the 4 raters (2 physicians and 2 physical therapists), the intraclass correlation coefficient (ICC) for interrater reliability for the total WGS score was excellent for the translated version of the instrument across the 2 times the instrument was administered (Day 1 ICC = 0.91; Day 2 ICC = 0.96). When examining the ratings of the 4 examiners for the individual items included in the WGS, values ranged from 0.91 to 1.00 indicating good agreement between the raters. The findings from the study point to the stability of the instrument when translated into another language and suggest that initially and with practice, multiple raters can use the instrument in a reliable manner. However, additional investigation of the WGS is warranted given the small sample size of the raters who participated in the study and the relatively short period of time between ratings.

Work by Turani et al. revealed fair to moderate correlations between the total WGS score upon admission and at discharge and with walking speed, the Functional Independent Measure™ and the Brunnstrom stages of motor recovery [39]. While a number of authors have concluded that the WGS was a valuable clinical measure [6, 32, 35, 39, 40], further investigation of its psychometric properties is necessary prior to use in actual clinical practice. One key step in demonstrating the validity and clinical utility of the WGS in actual clinical practice is to first establish its reliability. An instrument that is not reliable cannot be valid or useful for making clinical decisions. To date, interrater reliability, using a larger pool of raters, the stability of the instrument over time when used repeatedly by the same raters, and minimal detectable change have not been fully investigated. The purposes of this study were to examine the interrater and intrarater reliability of the WGS and to determine minimal detectable change when used by physical therapists to examine gait in individuals post-stroke. Minimal detectable change is the capacity of an instrument to identify changes in performance that are not due to measurement error. The videotaped performance of 6 participants post-stroke was used to determine reliability to minimize the effects of fatigue and recovery on the raters’ use of the WGS.

## Methods

### Participants

Fourteen licensed physical therapists, who worked full-time in 3 different acute inpatient rehabilitation centers, participated in the study as raters. Study enrollment required a minimum of 1 year of full-time clinical experience. This timeframe was selected to ensure that the raters had some experience examining gait in individuals with health conditions that affected walking. The exclusion criteria included prior experience using the WGS to examine gait and/or working with any of the participants post-stroke that were videotaped for the study. Prior experience with using the tool and familiarity with the participants post-stroke could have biased the results.

Six participants (mean age = 70.33 ± 11.72 years; age range = 51–82 years) with chronic stroke (5–106 months) agreed to help with the study by allowing the investigators to videotape them walking on a level surface. To be included in the study, the participants post-stroke had to be able to walk without any physical assistance, which would affect the qualitative gait parameters being measured by the WGS. Participants post-stroke were excluded if physical assistance was required at any time during the walking trials and if they were not medically stable to walk a minimum of 10 meters. Based on self-report, the participants post-stroke were all independent with ambulation in their home and could independently access the community. All had residual hemipareses and unilateral motor disturbance that affected gait. The study was reviewed and the solicitation of all participants was approved by the Widener University Institutional Review Board. All study participants provided signed informed consent prior to being enrolled in the study.

### Procedure

Videotaped gait performance was used to eliminate the effects of fatigue or recovery as factors affecting WGS reliability. Four cameras mounted on separate rolling dollies and tripods allowed the simultaneous recording of right and left lateral, anterior, and posterior views of each trial of the participants walking at preferred or usual speed along a 7.62 m pathway. The cameras providing the anterior and posterior views were mounted on fixed tripods and were zoomed manually as the participants post-stroke walked to allow continuous capture of the full body image. Cameras mounted on rolling dollies to provide lateral views were moved with the participant during the ambulation trial to decrease parallax and allow optimal viewing of gait kinematics, and spatial and temporal parameters. The individuals post-stroke performed 4 ambulation trials, walking at their preferred gait speed and using their usual assistive and orthotic devices. From the 4 trials completed, a single videotaped ambulation trial with the best visual quality was selected by the investigators for review for gait analysis by the physical therapists. The trial selected for rating ensured a full and close-up view of the entire person walking and allowed a visualization of the relevant joint segments for the items being scored by the WGS.

The single trial used for review provided anterior, posterior and lateral views of the individual walking. Each tape created contained 24 presentations of the individual post-stroke walking on a level surface – 6 anterior and posterior views and 6 views from the right and left side. The videotapes were created so that once started, each tape ran until all 24 presentations of the participant post-stroke walking were viewed. The appearance of the participants post-stroke on the tapes were organized to allow the raters to initially view the participant post-stroke walking 12 times – 3 consecutive anterior and posterior views and 3 consecutive views from the right and left sides or lateral views. This presentation was immediately followed by an area of the tape that was blank and lasted 2 min; the time was provided to allow scoring of the WGS by the raters. Finally, each tape conclude with an additional 12 ambulation trials being presented – 3 consecutive anterior and posterior views and 3 consecutive views from the right and left sides or lateral views.

On the first day of the study, the raters were given time to review the WGS and its scoring criteria before using the instrument to rate the videotapes of the individuals post-stroke. This approach was used to replicate what might happen in actual clinical practice where the scale would be used after a brief familiarization period and an opportunity to practice using the instrument. For the examination of interrater reliability, the viewing order was randomly predetermined and held constant across the 3 inpatient rehabilitation settings where the study was conducted. Each videotape containing the single ambulation trial was reviewed one time by the raters. The physical therapist had the opportunity to see the each of the views of the participant post-stroke walking. After watching the participant post-stroke walk 12 times, the raters were given 2 min to score the participant’s performance and then the same tape was re-played one more time to assist with finalizing the scoring. No specific instructions were given as to a particular approach to scoring the WGS. The raters had the option to score the WGS in real time, i.e. as they were viewing each of the tapes. The option to score the WGS during the blank portion of tape was also available. The final presentation of the participant post-stroke provided an additional opportunity to score the WGS or verifying scoring from the prior presentation of the person walking. The rating forms were collected after each videotape was reviewed. The videotapes were scored using the WGS with multiple raters in the same room simultaneously viewing the screen. However, discussion of the findings from each videotape was not allowed. The video of the participants walking was shown using a projector and a portable projection screen measuring 1.52 m in height and width.

Three weeks later, the original group of raters reviewed the videotapes a second time to establish intrarater reliability. Three weeks was thought to provide adequate time to prevent recall bias and ensured that the administration of the instruments across time was independent. Results from the prior administration of the WGS were not made available to the raters. The order in which the tapes were viewed was once again randomized and shown in the same sequence at each of the 3 inpatient rehabilitation settings. During the three-week period between the sessions, the raters were asked to not discuss the persons seen on the videotapes and the WGS, and to not practice using the WGS with actual patients. The investigators and raters followed the same sequence of events as the first session when using the WGS to rate the videotapes. The instructions were the same as the first session.

### Outcome measures

**Fig. 1:**
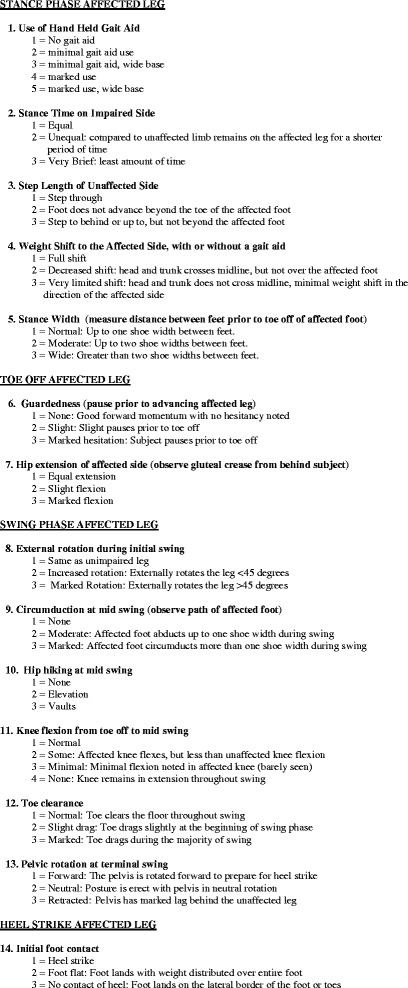
Wisconsin Gait Scale

The WGS was developed to specifically examine gait in individuals who have had a stroke [32]. The tool is comprised of 14 items that measure clinically relevant temporal and distance gait parameters and kinematics that are frequently altered after a stroke (Fig. 1). The first 7 WGS items examine the stance phase of gait focusing on the use of a hand held gait aid, stance time on the impaired side, step length of the unaffected side, weight shift to the affected side with or without gait aid and stance width. This portion of the scale also examines toe off of the affected leg, guardedness, and hip extension on the affected side. The WGS also examines 6 possible deviations of the affected limb during the swing phase of gait. Items 8 through 13 examine external rotation during initial swing, circumduction at mid swing, hip hiking at mid swing, knee flexion from toe off to mid swing, toe clearance, and pelvic rotation at terminal swing, respectively. The final item on the WGS examines ankle dorsiflexion at the initial contact phase of gait. A total summative score, which can range from 13.35–42.0, was calculated for the items. Items 2–10 and 12–4 are summed. Items 1 and 11 both contribute to the summary score but each is weighted or multiplied by ^3^/_5_ and ¾ respectively before being included in the final score [32]. Lower scores indicate better gait performance and fewer gait deviations.

### Statistical analysis

The two-way mixed Intraclass Correlation Coefficient (ICC) for absolute agreement for single measures was used to establish interrater [ICC_3,1_] and intrarater [ICC_2,1_] reliability [41, 42]. The ICC is a reliability coefficient that examines the effects of systematic and random errors on measurement repeatability; its values range from 0.00–1.00 [43]. Reliability was considered poor for ICC values less than 0.40, fair for values between 0.40 and 0.59, good for values between 0.60 and 0.74, and excellent for values between 0.75 and 1.00 [44]. In addition to the traditional way of understanding agreement using ICCs, there is the suggestion that the ICC for reliability should be greater than 0.90 to ensure reasonable validity for making clinical decisions related to individual performance and at least equal to 0.75 for making decisions about groups [45]. Finally, a *T*-test was used to examine differences between the ratings used to establish intrarater reliability.

Minimal detectable change (MDC) was estimated based on the 95 % confidence interval (CI), where MDC_95_ = standard error of the measurement x √2 × 1.96 [42]. MDC_95_ represents the magnitude of real change between measurements necessary to exceed error and measurement variability. The 95 % CI for MDC was calculated using 1.96 as the two sided z value and the √2 to account for the variance of the two measurements. The standard error of the measurement (SEM) was determined using the following equation, SEM = SD × √(1-r), where r was the reliability coefficient in the form of the ICC for intrarater reliability [43]. SEM, which is an indicator of absolute reliability, reports error in the same units as the measurement and can cancel out individual variations in measurement used to calculate the ICC [46]. SPSS Version 17.0 was used to conduct the statistical analyses (Chicago, IL: SPSS, Inc.).

Agreement between the measurements across the two sessions was also examined using Bland-Altman plots to identify mean difference between ratings and the 95 % confidence interval for the limits of agreement using MedCalc version 13.2 (MedCalc Software, Ostend, Belgium, (www.medcalc.org) [46].

## Results

The mean number of years of clinical experience for the physical therapists was 5.92 years (SD = 4.19, range = 1–16 years) and the average time worked at the inpatient rehabilitation settings where the study was conducted was 3.7 years (SD = 3.4, range = 0.04–12.5 years). All indicated having experience using OGA in clinical practice.

The average age of the participants post-stroke was 70.3 years (SD = 11.7) and 4 of the 6 had a single, unilateral left-sided stroke affecting the right side of the body, while the other 2 had a right-side stroke resulting in left-sided hemiparesis (Table 1). Two of the participants were female and 4 were male. The mean time post-stroke was 31.2 months (SD = 38.3, range 5–106 months). Four of the 6 participants post-stroke had Functional Ambulation Classification of 6 indicating the capacity for independent ambulation within the home and community across a variety of surfaces such as steps, inclines and uneven surfaces [46]. The remaining participants had lower Functional Ambulation Classifications of 4 and 5 due to limitations in the ability to independently manage steps and walk on uneven surfaces (Table 1). Average gait velocity for the sample was 0.57 m/sec (SD = 0.26, range 0.30–0.91).

**Table 1:** Participants post-stroke demographics

Demographic	Participant					
	1	2	3	4	5	6
Age	51	67	80	65	82	77
Gender	Male	Male	Male	Male	Female	Female
Side of lesion	Left	Left	Right	Right	Left	Left
Months post-stroke	25	5	34	106	9	8
Assistive device use	Yes	No	Yes	Yes	No	No
Functional ambulation classification	6	6	5	6	4	6
Mean Gait velocity (m/sec)	0.73	0.91	0.33	0.41	0.30	0.76

Approximately 10 min was required to review and score each tape. Interrater reliability was excellent, ICC_3,1_ = 0.83 and the 95 % CI ranged from 0.63–0.97. Table 2 summarizes (means and standard deviations) how the examiners rated each of the videos across the two sessions. The overall intrarater reliability was excellent, ICC_2,1_ = 0.91 and the 95 % CI ranged from 0.85–0.94. Across the ratings on day 1 and day 2, there were no statistically significant differences, t = 0.86, p = 0.40, mean difference = 0.65, and the lower and upper bounds of the 95%CI of the mean difference = −0.86 and 2.17, respectively.

**Table 2:** Mean Wisconsin Gait test scores by examination session occurring on 2 separate occasions, Intraclass Correlation Coefficient (ICC) for intrarater reliability, Standard Error of the Mean (SEM) and Minimal Detectable Change (MDC)

Videotape	Session 1	Session 2	ICC_2,1_	SEM	MDC
Participant 1	20.71 (2.51)	20.14 (2.06)	0.91 (0.85, 0.94)	1.47	4.24
Participant 2	14.73 (0.95)	14.35 (1.24)			
Participant 3	29.43 (2.02)	29.20 (2.15)			
Participant 4	20.48 (1.97)	19.06 (1.99)			
Participant 5	23.36 (2.60)	23.51 (1.87)			
Participant 6	23.75 (2.54)	22.27 (3.10)			

Sessions 1 & 2: values in parentheses represent standard deviation

*ICC* Intraclass Correlation Coefficient; values in parentheses indicate the 95 % Confidence interval, *SEM* Standard Error of the Measurement, *MDC* Minimal Detectable Change

The SEM, which identifies the amount of error present in the measurement, was 1.47 points. The measurements recorded were relatively stable across the time period for the ratings based on the means found for each of the videotapes (Table 2). The MDC_95_, or the smallest difference that can be detected by the WGS and not be attributable to chance variation and measurement error, was 4.24.

**Fig. 2:**
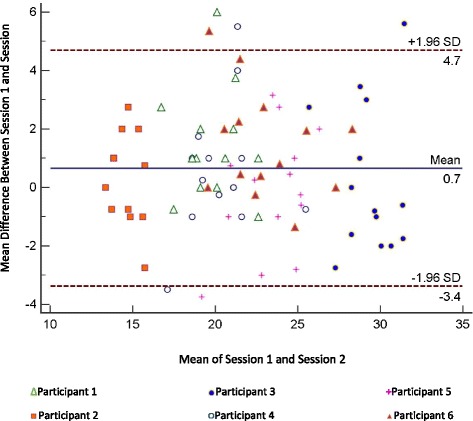
Bland-altman plot for intrarater reliability comparing the mean and differences between session 1 and session 2 showing the ratings for each of the videotapes

The findings from the Bland-Altman plot for each of the videotapes rated is summarized in Fig. 2. The mean difference between the measurements recorded at each session was relatively small, 0.65 (SD = 2.06) and the 95 % CI for the limits of agreement was −3.38 and 6.69 with only a small number of values falling outside that range. There did not appear to be systematic bias across the scores of the participants post-stroke within the range of scores extending from the lower end of the instrument to a score of 32–33.

## Discussion

The present study examined interrater and intrarater reliability of the WGS in a manner that, in part, replicates how tests and measures are typically used in actual clinical practice. Interrater and intrarater reliability were adequate to support clinical use. The WGS was stable over time for administration by the same raters based on the small differences between the repeated measurements, which on average was less than 1 point, and the calculated SEM (Table 2). Repeated administration of the WGS by the same group of raters was slightly higher on average. The high, intrarater reliability demonstrates the stability of the WGS in documenting walking performance over time when used by the same rater. Based on the notion that the ICC for making clinical decisions at an individual level should be higher than 0.90, the same rater using the instrument to repeatedly measure the same patient should provide useful information about which to make clinical decisions.

After a relatively brief period of time to review the instrument, the WGS was successfully used by the physical therapists to examine the gait of individuals post-stroke. As would commonly occur in clinical practice, reliability calculations were based on a single observation of individual performance, which in actual clinical practice would involve watching the individual walk several times from multiple perspectives.

Two factors known to contribute to measurement variability and error are instrument design and the examiner’s use of the instrument [46]. For the current study, the use of videotaping minimizes errors associated with individual performance. Multiple ambulation trials would be required for clinicians to complete the WGS in actual clinical practice. The need to view a walking performance multiple times can cause fatigue and performance variability. However, videotaping performance for the purposes of analysis can improve the reliability of OGA [25, 47] and is an option that may be readily available to physical therapists in clinical practice given the current state of technology.

This the first study to examine and report MDC for the WGS. MDC is one indicator of the confidence that differences in performance are not due to measurement error or patient variability. Therefore, an awareness of the number that indicates significant change in individual function is important to clinical practice. Increases or decreases in the score on the WGS that exceed MDC can indicate the need for services, provide justification for continued care or identify appropriate end points for care. In addition, MDC can assist with goal setting during an episode of care.

Interrater reliability was also good based on the ICC, which suggests that the WGS has the capacity to be used by multiple examiners to document gait dysfunction in individual post-stroke. In clinical practice, more than one health professional may be involved with treating an individual post-stroke, as the person moves across multiple settings during the recovery process. Implementing an outcome measure that is stable over time and capable of being used in a reliable manner by multiple therapists helps to objectively identify intervention effectiveness. However, the findings suggest caution when multiple raters plan on using the WGS given the variability in scores between raters for the Bland-Altman plots. The spread in the data could reflect the clinical experience of the participants, which has been suggested as being a factor affecting the reliability of OGA [25]. One solution that can be implemented to ensure multiple raters can effectively use the instrument is training and additional practice. The opportunity for extended practice and use of the instrument was not available to the raters in the current study. Using a smaller pool of raters, Yaliman and colleagues found higher ICC values [38].

When compared to work completed by Yaliman and colleagues, who reported interrater ICC values of 0.91 and 0.96, the value found in the current study was lower [38]. However, a variety of factors may account for the difference. Yaliman et al. had fewer raters, 2 physicians and 2 physical therapist, involved in the study and data collection occurred across 2 sessions separated by a period of only 2 days [38]. Thus, the difference between the 2 studies may reflect the experience of a limited number of examiners and their interpretation of the WGS scoring criteria. The timeframe between the 2 measurements in the Yaliman et al. study may also result in recall bias. Additional opportunities to practice using the instrument would likely improve interrater reliability, which is an outcome seen in the study conducted by Yaliman et al., where the second session interrater ICC value was higher [38]. Based on the literature, the reliability findings in the current study clearly exceeded that reported by most others using only OGA to examine gait in individuals post-stroke [31, 32, 47, 48].

The time required to complete the outcome measure is a consideration in the selection of an instrument to document walking kinematics after a stroke. Ferrarello and colleagues suggest that the Gait Assessment and Intervention Tool (GAIT) might be a better alternative to the WGS based on the total number of items included in the instrument and the research conducted to date demonstrating its reliability and responsiveness [31, 48, 49]. In examining the literature and considering issues affecting clinical utility, such as the amount of time required to complete the outcome measure, the WGS has fewer items, while demonstrating similar interrater reliability as the GAIT scale (ICC = 0.83) [48]. Videotaping of the individual post-stroke is required when using the GAIT scale and multiple ambulation trials are necessary to capture kinematic changes that appear in the different anatomical planes of movement. This requirement increases the administrative burden, which may make it less likely to be used in actual clinical practice. Videotaping walking, while improving OGA reliability, increases the amount of time required to gather the necessary data about function. Having fewer items may make the need for videoing the person walking either unnecessary or optional.

A concern has been raised about the lack of and/or number of items in the WGS directly measuring the kinematics of pelvis, knee and ankle during the stance phase of gait [31]. The items that comprise the instrument have the capacity to indirectly capture deficits affecting those joints or body limb segments. Items 2 and 3, which examine stance time and step length, indirectly capture kinematic changes occurring at the pelvis, hip, knee and ankle; item 14 examines ankle dorsiflexion kinematics at the initial contact phase of gait. The WGS includes items addressing spatial-temporal and kinematic gait parameters that are immediately relevant to the changes commonly, and perhaps easily observed after a stroke. This may account for the high interrater and intrarater reliability. The current study demonstrates the instrument’s clinical utility because it can be completed in less than 10 min. Incorporating more items may not necessarily provide better information on which to base clinical decisions about care. The administrative burden of the GAIT, which includes the number of items within the instrument and the need to videotape walking, may make this a more useful tool for research. Having fewer items, a reduce burden to administer the test and adequate psychometrics may make it more likely for clinicians to adopt the WGS as a measure to standardize OGA in clinical practice. With additional work exploring the psychometric properties of the WGS in a sample that is in the more acute phase of recovery, its usefulness for clinical practice may be further demonstrated. The lack of research investigating the psychometric properties of all available tools developed specifically to document gait dysfunction after a stroke remains a concern [31].

Outcome measures should be selected for clinical practice based on a sound understanding of their psychometric properties. OGA remains common in clinical practice [25, 30] and the use of standardized assessment instruments, such as the WGS, can improve both reliability and validity [31]. Documenting the changes in kinematic and spatial gait parameters using the WGS provides support for the effectiveness of interventions designed to improve the quality of walking after stroke. Changes in gait kinematics after a stroke affects oxygen consumption during walking and results in higher energy expenditure [50, 51]. The increased energy cost associated with participation in usual activities of daily living may account for participation restrictions occurring after a stroke. Interventions designed to alter post-stroke gait kinematics can contribute to faster walking speed, which is associated with increased gait efficiency [52–56]. The items in the WGS (Fig. 1) capture kinematic, temporal and spatial features of gait that contribute to slow walking speeds and increased energy expenditure [7, 54, 56] and correlate with motor recovery [57].

The WGS can complement other measures such as gait speed, which has been shown to be both reliable and valid for individuals post-stroke and linked to a number of functional outcomes [58, 59]. Changes in walking speed after a stroke can be connected to or explained by improved gait kinematics [19] and the WGS may be able to identify the specific areas of walking kinematics that were improved by selected interventions, The WGS provides a valuable and complementary understanding of walking performance that is not possible by only examining gait speed. Reductions in temporal and spatial gait asymmetries is associated with recovery and increased walking stability after a stroke [6, 60].

### Study limitations

There are a number of limitations that should be considered when applying the results either for the purposes of research or using the findings to make decisions affecting clinical practice. The use of videotapes had an effect on the reliability coefficients and the score identifying minimal detectable change. Videotaping eliminated one source of measurement variability arising from physiological factors associated with the need to have the participants post-stroke walk multiple times. With any standardized instrument, 3 factors can affect reliability – the patient or individual post-stroke and their performance, the instrument and the rater. Videotaping those post-stroke, reduced or eliminated the first of the 3 factors and allowed a judgments to made about the reliability of the instrument based on the capacity of the raters to effectively used the WGS and the understandability of the items and scoring instructions contained within the instrument. The study design was purposely selected to curtail the effects of fatigue, which allowed the best investigation of the psychometric properties of the WGS. For the purposes of this study and the number of physical therapists who participated on multiple occasions and in multiple locations, reviewing videotapes was the only available option. Fatigue may play a role in making clinical decisions about gait function and the option to videotape a walking provides a way not only to enhance the reliability and validity of observational gait analysis but also creates an additional record of the individual’s performance. In a clinical setting, physical therapists make choices about how to report patient performance (best versus worst) based on having ongoing opportunities for contact with the patient.

Given the sample, the findings are best applied to individuals in the chronic or sub-acute phases of stroke recovery. All study participants post-stroke, while having residual motor deficits that were captured by the WGS, were independent for household ambulation. Future research will need to examine a patient population that is in the acute phase of recovery and less functionally independent with ambulation. For those post-stroke, who are in the acute phase of recovery, gait deviations may be more obvious and the WGS may demonstrate even greater reliability.

Finally, for a study of reliability, the sample size is relatively small both for the number of participants who were physical therapists and those post-stroke. Limiting the number of participants post-stroke and videotaping walking allowed a greater number of physical therapists to be involved in the study and ensured the stability of walking performance. The findings from the current study provide insight into how much variability may be present due to rater and instrument factors and suggest that the instrument has promise as a tool to assist with OGA in those post-stroke.

## Conclusions

The WGS appears to be a useful clinical outcome measure that can improve the reliability and objectivity of OGA. The results of this study confirms the interrater and intrarater reliability of the WGS when administered by physical therapists to examine gait in individuals post-stroke and contributes to further the understanding of the psychometric properties of instrument. The study also provided values to determine when significant clinical change has occurred if the scale is used in clinical practice or for the purposes of research.

## Abbreviations

CI: Confidence interval
CVA: Cerebrovascular accident
MDC: Minimal detectable change
OGA: Observational gait analysis
WGS: Wisconsin gait scale

## References

[1] Sullivan KJ, Cen SY (2011). Model of disablement and recovery: knowledge translation in rehabilitation research and practice. Phys Ther.

[2] Daly JJ, Roenigk K, Cheng R, Ruff RL (2011). Abnormal leg muscle latencies and relationship to dyscoordination and walking disability after stroke. Rehabil Res Pract.

[3] Arene N, Hidler J (2009). Understanding motor impairment in the paretic lower limb after a stroke: a review of the literature. Top Stroke Rehabil.

[4] LeBrasseur NK, Sayers SP, Ouellette MM, Fielding RA (2006). Muscle and behavioral factors mediate functional limitations and disability following stroke. Phys Ther.

[5] Kluding P, Gajewski B (2009). Lower extremity strength differences predict activity limitations in people with chronic stroke. Phys Ther.

[6] Patterson KK, Parafianowicz I, Danells CJ, Closson V, Verrier MC, Staines WR (2008). Gait asymmetry in community-ambulating stroke survivors. Arch Phys Med Rehabil.

[7] von Schroeder HP, Coutts RD, Lyden PD, Billings E, Nickel VL (1995). Gait parameters following stroke: a practical assessment. J Rehabil Res Dev.

[8] Goldie PA, Matyas TA, Evans OM (2001). Gait after stroke: Initial deficit and changes in temporal patterns for each gait phase. Arch Phys Med Rehabil.

[9] Bensoussan L, Mesure S, Viton JM, Delarque A (2006). Kinematic and kinetic asymmetries in hemiplegic patients’ gait initiation patterns. J Rehabil Med.

[10] Roth EJ, Merbitz C, Mroczek K, Dugan SA, Suh WW (1997). Hemiplegic gait: relationships between walking speed and other temporal parameters. Am J Phys Med Rehabil.

[11] Reisman DS, Rudolph KS, Farquhar WB (2009). Influence of speed on walking economy poststroke. Neurorehabil Neural Repair.

[12] Platts MM, Rafferty D, Paul L (2006). Metabolic cost of overground gait on younger stroke patients and healthy controls. Med Sci Sports Exerc.

[13] Forster A, Young J (1995). Incidence and consequences offalls due to stroke: a systematic inquiry. Br Med J.

[14] Weerdesteyn V, de Niet M, van Duijnhoven HJ, Geurts AC (2008). Falls in individuals with stroke. J Rehabil Res Dev.

[15] Jette DU, Latham NK, Smout RJ, Gassaway J, Slavin MD, Horn SD (2005). Physical therapy interventions for patients with stroke in inpatient rehabilitation facilities. Phys Ther.

[16] Tyson S, Watson A, Moss S, Troop H, Dean-Lofthouse G, Jorritsma S (2008). Development of a framework for the evidence-based choice of outcome measures in neurological physiotherapy. Disabil Rehabil.

[17] Kaczmarczyk K, Wit A, Krawczyk M, Zaborski J, Gajewski J (2012). Associations between gait patterns, brain lesion factors and functional recovery in stroke patients. Gait Posture.

[18] Bonnyaud C, Pradon D, Zory R, Bensmail D, Vuillerme N, Roche N (2013). Does a single gait training session performed either overground or on a treadmill induce specific short-term effects on gait parameters in patients with hemiparesis? A randomized controlled study. Top Stroke Rehabil.

[19] Reisman D, Kesar T, Perumal R, Roos M, Rudolph K, Higginson J (2013). Time course of functional and biomechanical improvements during a gait training intervention in persons with chronic stroke. J Neurol Phys Ther.

[20] Nadeau S, Duclos C, Bouyer L, Richards CL (2011). Guiding task-oriented gait training after stroke or spinal cord injury by means of a biomechanical gait analysis. Prog Brain Res.

[21] Yavuzer G, Geler-Kulcu D, Sonel-Tur B, Kutlay S, Ergin S, Stam HJ (2006). Neuromuscular electric stimulation effect on lower-extremity motor recovery and gait kinematics of patients with stroke: a randomized controlled trial. Arch Phys Med Rehabil.

[22] Davis RB (1997). Reflections on clinical gait analysis. J Electromyogr Kinesiol.

[23] Toro B, Nester CJ, Farren PC (2003). The status of gait assessment among physiotherapists in the United Kingdom. Arch Phys Med Rehabil.

[24] Krebs DE, Edelstein JE, Fishman S (1985). Reliability of observational kinematic gait analysis. Phys Ther.

[25] Eastlack ME, Arvidson J, Snyder-Mackler L, Danoff JV, McGarvey CL (1991). Interrater reliability of videotaped observational gait-analysis assessments. Phys Ther.

[26] Williams G, Morris ME, Schache A, McCrory P (2009). Observational gait analysis in traumatic brain injury: Accuracy of clinical judgment. Gait Posture.

[27] Brunnekreef JJ, van Uden CJ, van Moorsel S, Kooloos JG (2005). Reliability of videotaped observational gait analysis in patients with orthopedic impairments. BMC Musculoskelet Disord.

[28] Coutts F (1999). Gait analysis in the therapeutic environment. Man Ther.

[29] Perry J, Burnfield JM (2010). Gait analysis : normal and pathological function.

[30] Toro B, Nester C, Farren P (2003). A review of observational gait assessment in clinical practice. Physiother Theory Pract.

[31] Ferrarello F, Bianchi VA, Baccini M, Rubbieri G, Mossello E, Cavallini MC (2013). Tools for observational gait analysis in patients with stroke: a systematic review. Phys Ther.

[32] Rodriquez AA, Black PO, Kile KA, Sherman J, Stellberg B, McCormick J (1996). Gait training efficacy using a home-based practice model in chronic hemiplegia. Arch Phys Med Rehabil.

[33] Algurén B, Fridlund B, Cieza A, Sunnerhagen KS, Christensson L (2012). Factors associated with health-related quality of life after stroke: a 1-year prospective cohort study. Neurorehabil Neural Repair.

[34] Balaban B, Tok F (2014). Gait Disturbances in Patients With Stroke. PM & R.

[35] Patil P, Rao S (2011). Effects of Thera-Band (R) elastic resistance-assisted gait training in stroke patients: a pilot study. Eur J Phys Rehabil Med.

[36] Onigbinde AT, Awotidebe T, Awosika H (2009). Effect of 6 weeks wobble board exercises on static and dynamic balance of stroke survivors. Technol Health Care.

[37] In Mo P, Duck Won O, Suhn Yeop K, Jong DC (2010). Clinical feasibility of integrating fast-tempo auditory stimulation with self-adopted walking training for improving walking function in post-stroke patients: a randomized, controlled pilot trial. J. Phys. Ther. Sci..

[38] Yaliman A, Kesiktas N, Ozkaya M, Eskiyurt N, Erkan O, Yilmaz E (2014). Evaluation of intrarater and interrater reliability of the Wisconsin Gait Scale with using the video taped stroke patients in a Turkish sample. NeuroRehabilitation.

[39] Turani N, Kemiksizoglu A, Karatas M, Ozker R (2004). Assessment of hemiplegic gait using the Wisconsin Gait Scale. Scand J Caring Sci.

[40] Pizzi A, Carlucci G, Falsini C, Lunghi F, Verdesca S, Grippo A (2007). Gait in hemiplegia: evaluation of clinical features with the Wisconsin Gait Scale. J Rehabil Med.

[41] Shrout PE, Fleiss JL (1979). Intraclass correlations: uses in assessing rater reliability. Psychol Bull.

[42] Weir JP (2005). Quantifying test-retest reliability using the intraclass correlation coefficient and the SEM. J Strength Cond Res.

[43] Haley SM, Fragala-Pinkham MA (2006). Interpreting change scores of tests and measures used in physical therapy. Phys Ther.

[44] Cicchetti D, Bronen R, Spencer S, Haut S, Berg A, Oliver P (2006). Rating scales, scales of measurement, issues of reliability: resolving some critical issues for clinicians and researchers. J Nerv Ment Dis.

[45] Portney LG, Watkins MP (2009). Foundations of Clinical Research: Applications to Practice.

[46] Atkinson G, Nevill AM (1998). Statistical methods for assessing measurement error (reliability) in variables relevant to sports medicine. Sports Med.

[47] McGinley JL, Goldie PA, Greenwood KM, Olney SJ (2003). Accuracy and reliability of observational gait analysis data: judgments of push-off in gait after stroke. Phys Ther.

[48] Daly JJ, Nethery J, McCabe JP, Brenner I, Rogers J, Gansen J (2009). Development and testing of the Gait Assessment and Intervention Tool (G.A.I.T.): A measure of coordinated gait components. J Neurosci Methods.

[49] Zimbelman J, Daly JJ, Roenigk KL, Butler K, Burdsall R, Holcomb JP (2012). Capability of 2 Gait Measures for Detecting Response to Gait Training in Stroke Survivors: Gait Assessment and Intervention Tool and the Tinetti Gait Scale. Arch Phys Med Rehabil.

[50] Detrembleur C, Dierick F, Stoquart G, Chantraine F, Lejeune T (2003). Energy cost, mechanical work, and efficiency of hemiparetic walking. Gait Posture.

[51] Reisman DS, Rudolph KS, Farquhar WB (2009). Influence of speed on walking economy poststroke. Neurorehabil Neural Repair.

[52] Lamontagne A, Fung J (2004). Faster Is Better: Implications for Speed-Intensive Gait Training After Stroke. Stroke.

[53] Patterson SL, Rodgers MM, Macko RF, Forrester LW (2008). Effect of treadmill exercise training on spatial and temporal gait parameters in subjects with chronic stroke: a preliminary report. J Rehabil Res Dev.

[54] Hall AL, Bowden MG, Kautz SA, Neptune RR (2012). Biomechanical variables related to walking performance 6-months following post-stroke rehabilitation. Clin Biomech..

[55] Sabut SK, Lenka PK, Kumar R, Mahadevappa M (2010). Effect of functional electrical stimulation on the effort and walking speed, surface electromyography activity, and metabolic responses in stroke subjects. J Electromyogr Kinesiol.

[56] Awad LN, Palmer JA, Pohlig RT, Binder-Macleod SA, Reisman DS (2015). Walking speed and step length asymmetry modify the energy cost of walking after stroke. Neurorehabil Neural Repair.

[57] Brandstater ME, de Bruin H, Gowland C, Clark BM (1983). Hemiplegic gait: analysis of temporal variables. Arch Phys Med Rehabil.

[58] Richards CL, Malouin F, Dean C (1999). Gait in stroke: assessment and rehabilitation. Clin Geriatr Med.

[59] Perry J, Garrett M, Gronley JK, Mulroy SJ (1995). Classification of walking handicap in the stroke population. Stroke.

[60] McAndrew Young PM, Dingwell JB (2012). Voluntarily changing step length or step width affects dynamic stability of human walking. Gait Posture.

